# Neuropsychiatric manifestations of systemic lupus erythematosus: Iranian experience

**DOI:** 10.4103/0972-2327.64633

**Published:** 2010

**Authors:** Afshin Borhani Haghighi, Shahab Ghasem Haza

**Affiliations:** Transgenic Technology Research Center and Departments of Neurology, Shiraz University of Medical Sciences, Shiraz, Iran; 1 Research Center for Traditional Medicine and History of Medicine, Shiraz University of Medical Sciences, Shiraz, Iran

**Keywords:** Classification, neurological, systemic lupus erythematosus

## Abstract

**Aims:**

To evaluate the prevalence and characteristics of different neurological and psychiatric presentations in patients admitted to hospital with systemic lupus erythematosus (SLE).

**Materials and Methods:**

In this retrospective hospital-based study, we examined the medical records of patients with SLE who were referred to the hospitals affiliated to Shiraz University of Medical Sciences from 1995 to 2005. All patients of SLE who had clinical neurological or psychiatric features were included in this study. The patients´ demographic data, findings on general examination, neuropsychiatric manifestations, and the results of laboratory investigations and imaging studies were recorded. Clinical manifestations were classified according to the American College of Rheumatology (ACR) case definitions.

**Results:**

Of the 407 study patients, 11.3% had neuropsychiatric complications. The most frequent findings were seizure (63%), headache (60%), and decreased level of consciousness (50%). Cerebrovascular disease (28.3%), seizure disorder (26.5%), and acute confusional state (19.6%) were the most prevalent syndromes.

**Conclusion:**

The prevalence and nature of different neurological presentations of SLE in Iranian patients has some similarities to that seen in other populations, as well as some differences. Ethnic and environmental factors may contribute to these differences.

## Introduction

Systemic lupus erythematosus (SLE) is a chronic autoimmune disease with a variable clinical presentation and a variable course. It involves almost every organ of the body. Diverse psychiatric and neurological disorders may complicate the course of the disease. The prevalence of nervous system involvement in SLE is reported to be 18–67%, depending on the diagnostic criteria used.[1-3] These manifestations can occur frequently, may be highly variable, and are often incapacitating and sometimes life threatening.

Central nervous system (CNS) manifestations include headache, seizure, transient ischemic attack, stroke, movement disorders, cerebral venous thrombosis, idiopathic intracranial hypertension, optic neuritis, cranial nerve palsies, cerebellar syndromes, transverse myelitis, and other rare neurological symptoms.[1-5] The psychiatric manifestations of SLE are also very variable and include amnesia, acute confusional state, delirium, dementia, depression, anxiety, obsessive-compulsive disorder, hallucinosis, and schizophrenia-like psychosis.[6,7]

Peripheral nervous system and muscular manifestations are less frequent than CNS symptoms and include different type of neuropathies, myasthenia gravis, Lambert-Eaton syndrome, inflammatory polymyositis, and orbital myositis.[8-10]

In addition to the primary neuropsychiatric manifestations of lupus, some complications of SLE may affect the CNS secondarily. Metabolic encephalopathies due to uremia, electrolyte disturbances, hypertensive encephalopathies, CNS infections, cardiac embolism, intracranial hemorrhage due to thrombocytopenia or coagulopathies, and side effects of drugs are some examples.

The etiopathogenesis of neuropsychiatric SLE is still obscure. The most probable pathomechanism is the existence of a vasculopathic state (abnormal vessel walls without inflammatory cell infiltrates) of small vessels which alters cerebral microcirculation. A true vasculitic process occurs much less often.[11]

Several structural, functional, and integrative investigations like autoantibody batteries, cerebrospinal fluid (CSF) analyses, computerized tomography (CT), magnetic resonance imaging (MRI), positron emission computerized tomography (PET), single photon emission computerized tomography (SPECT), magnetic resonance spectroscopy, diffusion-and perfusion-weighted imaging, magnetization transfer imaging, and electrodiagnostic studies are all used to study neurological involvement in SLE, but no single tool has proven to be completely specific and sensitive.[12]

Because very little is known about the frequency of the neurological and psychiatric manifestations of SLE in Iran, this study was done to evaluate the frequency and characteristics of these manifestations in Iranian patients with SLE.

## Materials and Methods

This is a descriptive retrospective study done to find out the frequency and characteristics of the neurological and psychiatric manifestations of SLE patients referred to the hospitals affiliated to the Shiraz University of Medical Sciences during a 11 year period from 1995 to 2005. The clinical records of 407 patients discharged from hospital after a diagnosis of SLE were reviewed. Complete confidentiality was maintained. The hospital files of the patients were examined for mention of the presence of neuropsychiatric symptoms and signs. Inclusion criteria were fulfillment of the ACR criteria for SLE and presence of a syndrome or disease with clinical neurological or psychiatric features likely to be causally related to SLE. The patients who had any clinical or paraclinical findings which were consistent with any clinical entity other than SLE were excluded from the study. These exclusion criteria included positive CSF cultures or any other evidence of neurological infections, diabetes mellitus, hyperlipidemia, positive family history of premature atherosclerosis or other major risk factors of young adult stroke, intravenous drug abuse, neurological side effects of drugs, hypertensive or uremic encephalopathy, electrolyte disturbances, complications of renal transplantation, and positive HIV test.

The patients' demographic data, duration of SLE, general manifestations of the disease, and the neurological and psychiatric manifestations of SLE were studied. The results of paraclinical data such as antinuclear antibodies, anti-double-strand DNA, CSF analyses, electroencephalography, nerve conductions studies and electromyography, and neuroimaging studies were also recorded from the medical files.

Cognitive dysfunction was considered according to clinical files of the patients.and comprehensive psychometric batteries were not conducted for most of the patients.

The patients were categorized by a neurologist (A.B.H) into definite neurological syndromes according to American College of Rheumatology (ACR) criteria.[13]

We used SPSS 10.0 software for analysis of the data. The Chi square test, the *t*-test, and Fisher's exact test were used as appropriate. Statistical significance was assumed at *P* ≤ 0.05.

## Results

Among the 407 patients, 59 had neurological or psychiatric symptoms. Thirteen patients were excluded due to the presence of one of the following: uremic encephalopathy resulting from renal involvement by SLE (seven patients), hypocalcemia, bacterial meningitis, thrombocytopenia-induced intracranial hemorrhage, hypertension-induced intracranial hemorrhage, cardiac embolism originating from a prosthetic valve, and idiopathic epilepsy (each in one patient). The remaining 46 patients (11.3% of the patient population) were considered to have neuropsychiatric lupus.

Among these 46 patients, 42 (91.3%) were female and four (8.7%) were male. Thirty-one patients (67.4%) were from urban areas and 15 patients (32.6%) from rural areas. The age at first manifestation of SLE ranged between 16 and 47 years, with a mean of 24 years. The mean age at first manifestation of neuropsychiatric lupus was 27 years, with a mean interval of 4 years between diagnosis of SLE and manifestation of neuropsychiatric symptoms/signs. In 11 patients (23%), neurological or psychiatric manifestations were the initial features of SLE.

A malar rash was the most frequent manifestation in the studied patients and was seen in 42 patients (91.3%). The other frequent manifestations were renal involvement (89%), serositis (68%), hematological disorders (63%), arthritis (50%), and photosensitivity (43%).

**Figure 1:**
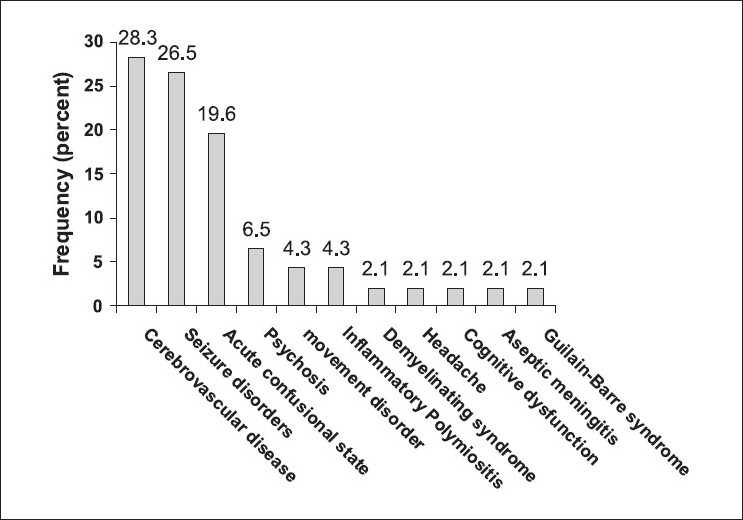
Frequency of the observed of SLE in the studied patients according to criteria of the American College of Rheumatology.[13] Inflammatory polymyositis is not an ACR entry

Among the neuropsychiatric manifestations in 46 patients, general seizures were the most frequent finding, followed by headaches, decreased level of consciousness, and weakness (including mono/hemi/para/quadriparesis) [Table 1]. Figure 1 shows the prevalence of different clinical syndromes among our patients according to ACR criteria. As can be seen, cerebrovascular diseases, seizure disorders, and acute confusional state were the 'big three' among our patients.

**Table 1:** Frequency of the observed neuropsychiatric manifestations of SLE in the studied patients

Manifestation	Frequency	(%)	Manifestation	Frequency	(%)
Generalized seizures	29	(63.0)	Numbness	3	(6.5)
Headaches	28	(60.1)	Papilledema	3	(6.5)
Decreased level of consciousness	23	(50)	Urinary incontinence	3	(6.5)
Weakness	14	(30.4)	Extremity pain	2	(4.3)
Nausea and vomiting	13	(28.2)	Insomnia	2	(4.3)
Hallucination	13	(28.3)	Ptosis and diplopia	2	(4.3)
Hyperrefl exia	12	(26.1)	Chorea	2	(4.3)
Blurred vision	10	(21.7)	Lhermitte sign	1	(2.1)
Dizziness	8	(17)	Neck pain	1	(2.1)
Major depression	8	(17)	Ataxia	1	(2.1)
Babinski's sign	8	(17)	Aphasia	1	(2.1)
Fatigue	5	(11)	Amaurosis fugax	1	(2.1)
Tremor	5	(11)	Hoffman sign	1	(2.1)
Facial palsy	4	(8.5)			
Dysphagia	4	(8.5)			

In this study, 92% of patients had high erythrocyte sedimentation rate (ESR). Antinuclear antibody (ANA) and anti-double-strand DNA were positive in 94% and 78% of patients, respectively. C3 and/or C4 complement were decreased in 68% of patients.

Thirty-nine CT scans and 33 MRIs were conducted in all. Among the CT scans of brains, 23 (59%) revealed no abnormality. In seven (18%), brain atrophy was present, five scans (13%) showed diffuse brain edema, and four scans (10%) showed large infarcts. Among the 33 available brain MRIs, 12 (36%) studies were normal, small lesions were seen in 17 (52%) brain MRIs, and large lesions were observed in four (12%) MRIs. All of the abnormal MRIs revealed hyperintense lesions in T2-weighted images but only in 9 (43%) MRIs were corresponding hypointense lesions in T1-weighted images seen. These characteristics were compatible with ischemic/demyelinating lesions.

There were 22 CSF analyses performed in the study subjects. Fifty-five percent of the CSF studies were completely normal, 27% revealed pleocytosis ( segment predominant in half and lymphocytic predominant in half), 18% showed increased protein content, and 2% showed decreased glucose levels.

Nerve conduction studies and electromyography revealed myogenic pattern in two patients; there was myositis and a neurogenic pattern with conduction block in one patient with the Guillain-Barré syndrome.

The mortality rate due to direct neuropsychiatric complications of SLE in our 46 patients was 22%. The major contributor to mortality was large cerebrovascular accidents, which were seen in 69% of the patients who died.

## Discussion

This is a retrospective hospital-based study, with data collected from clinical files of patients. Therefore, patients with non-catastrophic neuropsychiatric manifestations of SLE were not included in our study, which had some influences on our results.

The prevalence of neuropsychiatric manifestations in our study population is lower than that reported in previous studies.[1-3] This could be because of the retrospective, hospital-based nature of our study, which failed to detect the cases of SLE with neurological manifestations that did not require hospitalization. The high mortality rate among our patients is also a consequence of the nature of selection.

In our study, like in many other studies, seizures were the most frequent neurological manifestations.[14-16] Seizures can occur at any time during the course of SLE and all kinds of seizures may be seen in these patients (including generalized tonic-clonic, simple focal, and complex partial seizures). They may occur in isolation or along with the other neurological manifestations.[14,17,18]

Sixty percent of our patients had headache as part of their neuropsychiatric event. According to previous reports the frequency of headache among SLE patients is 28–68%.[19-21]

The increased frequency of cerebrovascular events among SLE patients has been evident both in large series and in epidemiological studies.[22-24] For example, the accumulated frequency of cerebrovascular disease (infarcts, transient ischemic attacks, and hemorrhages) was 23.1% according to Futurell and Milikan.[22]The possible pathomechanisms are premature atherosclerosis, vasculitis, and coagulopathy.[23]

Psychotic attacks are relatively common in SLE and sometimes recur. Psychotic attacks in SLE must be differentiated from corticosteroid-induced psychosis.[25]

Movement disorders which occur in SLE include chorea (which was seen in two of our patients),[26] ballismus,[27] and parkinsonism.[28]

Cerebral venous thrombosis presents with severe headache, papilledema, and/or hemorrhagic infarcts and was seen in one of our patients. Due to the availability of magnetic resonance venography the diagnosis of venous sinus thrombosis in patients with lupus is relatively easy these days.[29]

Although inflammatory polymyositis is not included in the ACR nomenclature system, we considered it as a separate entity in this study so as to cover all the neurologic complications of SLE.

There are several series which have used the ACR nomenclature for categorization of neuropsychiatric manifestations of SLE. In Brazilian and Thai series,[15,16] seizure disorder was the most common syndrome (whereas it was the second most common one in our study). Cognitive dysfunction, one of the least common syndromes in our study, was one of the most common ACR syndromes in American,[30] Finnish,[31] and Italian[32] studies. This variation may be due to not only geographic and ethnic factors but also to the variations in the selection criteria in the different studies.

The current study has some shortcomings, the major limitations being its retrospective and hospital-based nature. Also, we had very strict exclusion criteria; If both SLE and its complications contributed to the neuropsychiatric manifestations in any one patient, exclusion of that patient may have induced a selection bias. Also considering a patient in just one ACR category might have been arbitrary.

In summary, our study restates the great diversity of neuropsychiatric manifestations in SLE. Based on our experience in this study, we stress the need for conducting a prospective multicentric study on neuro-lupus, with inclusion of both inpatients and outpatients.
